# Preparative Separation of Alkaloids from Stem of *Euchresta tubulosa* Dunn. by High-Speed Counter-Current Chromatography Using Stepwise Elution

**DOI:** 10.3390/molecules24244602

**Published:** 2019-12-16

**Authors:** Weixin Li, Huan Wang, Aiwen Dong

**Affiliations:** Key Laboratory of Hunan Forest Products and Chemical Industry Engineering, Jishou University, Zhangjiajie 427000, China; liweixin@stu.jsu.edu.cn (W.L.); wh120108@163.com (H.W.)

**Keywords:** alkaloids, *Euchresta tubulosa* Dunn., chemical structures, high-speed counter-current chromatography, stepwise elution

## Abstract

*Euchresta tubulosa* Dunn. is a Chinese herbal medicine with biological activity, but there are few studies on its components at present. Alkaloids in the stem of *Euchresta tubulosa* Dunn. were isolated and purified by high-speed counter-current chromatography (HSCCC) using stepwise elution. First of all, liquid-liquid extraction (methylene chloride-methanol-water, 5:1:4, *v*/*v*) was used for the preliminary enrichment. According to the partition coefficient (*K*) of a target compound in a series of different two-phase solvents, the final result was that carbon tetrachloride-methylene chloride-methanol-water (2:3:3:2, *v*/*v*) (1) and methylene chloride-methanol-water (5:3:2, *v*/*v*) (2) were suitable for the HSCCC using stepwise elution. As a result, the purity was all higher than 93% and matrine (**1**), oxymatrine (**2**), *N*-formyl cytisine (**3**), and *N*-acetyl cytisine (**4**) can be eluted at one time by this mode. Cytisine-type alkaloids were isolated for the first time in this plant. Finally, the applicability of the mode was verified.

## 1. Introduction.

*Euchresta tubulosa* Dunn. (Leguminous, Euchresta) is the evergreen shrub mainly distributed in Sichuan, Chongqing, Hunan, and Hubei [1]. It is also known as Odougen, Hudoulian, Dougan, and Opium Seven in China. It contains many alkaloids that have a number of biological activities [2], such as inhibiting the proliferation of human hepatocellular carcinoma cells [3], alleviating tissue injury, promoting autophagy in ischemia-reperfusion rats [4], and other biological activity [5,6]. The common methods for the separation and purification of alkaloids are silica gel column chromatography, thin-layer chromatography, Sephadex LH-20, and recrystallization [7,8], which have the disadvantages of a long cycle, complex steps, high solvent consumption, etc.

High-speed counter-current chromatography (HSCCC) is a preparative all-liquid partition chromatography technique without using a solid carrier, first introduced by Ito [9] in the early 1980s. HSCCC can effectively eliminate the irreversible adsorption of samples on the solid carrier. Therefore, it is very suitable for the separation and purification of alkaloids [10], coumarins [11,12], flavonoids [13], anthraquinones [14], and polyphenols [15] from natural products. In HSCCC, the mobile phase with different polarity is often used for stepwise elution [16,17], in order to separate quickly and effectively.

The aim of this study is to establish a mode for sample pretreatment and one-time elution of alkaloids with different polarities. The accidental discovery of the solvent system of this mode was due to the earlier work of fractions A and B by silica gel column chromatography. The purity and structure of the monomer were analyzed, and the applicability of the model was verified.

## 2. Results

**Figure 1:**
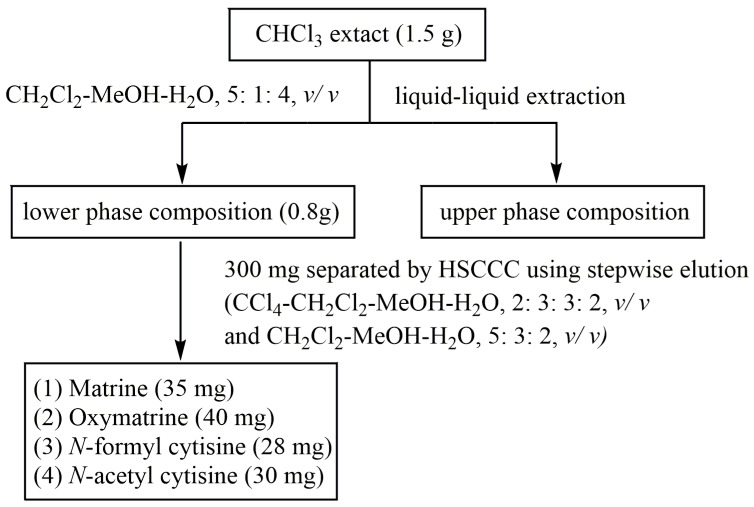
Optimized process flow chart.

This model is based on a previous study of silica gel column chromatography, combined with the current HSCCC separation technology, which can be used to separate and purify alkaloids with a similar structure. The model is shown in Figure 1.

### 2.1. Selection of Optimum Solvent Systems of HSCCC Separation

The *K* of the target compound was calculated according to Formula (1) and HPLC-UV. Table 1 shows the *K* of the four target compounds.

The two-phase solvent system of fraction A with a bigger R_f_ was screened by the Ito method [18]. Firstly, this experiment began with CH_2_Cl_2_-MeOH-H_2_O (2:1:1, *v*/*v*). The proper *K* can be obtained by changing the volume ratio of the solvent and adding carbon tetrachloride in the system. Table 1 shows the *K* values. According to the actual situation and HSCCC operation, CCl_4_-CH_2_Cl_2_-MeOH-H_2_O (2:3:3:2, *v*/*v*) was determined as the optimum solvent system of fraction A.

The two-phase solvent system of fraction B with a smaller R_f_ was screened by the Ito method, too. Table 1 shows that *K*_3_ and *K*_4_ are suitable in CH_2_Cl_2_-MeOH-H_2_O (2:1:1, *v*/*v*). Minor adjustments were made according to the actual situation and HSCCC operation. Finally, CH_2_Cl_2_-MeOH-H_2_O (5:3:2, *v*/*v*) was determined to be the optimal solvent system of fraction B.

It was found that the polarities of the two fractions were quite different. However, the stationary phase of the two solvent systems was the same. Therefore, preparative separation of four alkaloids by HSCCC using stepwise elution was considered.

### 2.2. Sample Pretreatment of HSCCC Separation

The results showed that four alkaloids were easily dissolved in dichloromethane. It was found that CH_2_Cl_2_-MeOH-H_2_O (5:1:4, *v*/*v*) can retain most of the four alkaloids in the lower phase and can remove the compounds with high water and methanol solubility. The crude alkaloid extract (1.5 g) was extracted until the dichloromethane layer almost had no precipitation reaction with bismuth potassium iodide. Finally, 0.8 g of the sample was obtained by combining the dichloromethane layer.

### 2.3. HSCCC Separation

**Figure 2:**
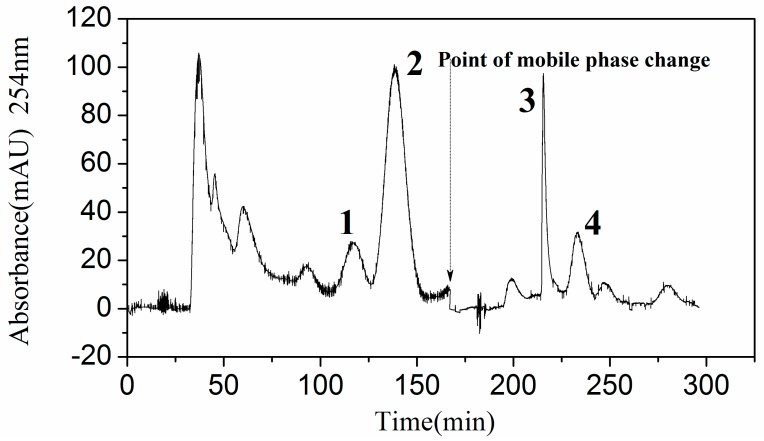
High-speed counter-current chromatography (HSCCC) of the sample by liquid-liquid partition. Solvent systems: CCl_4_-CH_2_Cl_2_-MeOH-H_2_O (2:3:3:2, *v*/*v*) and CH_2_Cl_2_-MeOH-H_2_O (5:3:2, *v*/*v*); mode: Stepwise elution; stationary phase: Upper phase of CCl_4_-CH_2_Cl_2_-MeOH-H_2_O (2:3:3: 2, *v*/*v*); mobile phase: Lower phase of the two solvent systems; flow rate: 1.8 mL/min; revolution speed: 900 rpm; temperature: 25 °C; sample size: 300 mg dissolved in equal volume of lower and upper phase (each 5 mL); retention of the stationary phase (S_f_): 72%.

Thus as to shorten the preparation time and reduce the workload, HSCCC using stepwise elution was adopted. Finally, both CCl_4_-CH_2_Cl_2_-MeOH-H_2_O (2:3:3:2, *v*/*v*) and CH_2_Cl_2_-MeOH-H_2_O (5:3:2, *v*/*v*) were selected for the isolation of four target alkaloids. The two alkaloids (matrine-type) were eluted by the lower phase of CCl_4_-CH_2_Cl_2_-MeOH-H_2_O (2:3:3:2, *v*/*v*), and then the two alkaloids (cytisine-type) were eluted by the lower phase of CH_2_Cl_2_-MeOH-H_2_O (5:3:2, *v*/*v*). Figure 2 shows the results of HSCCC with stepwise elution; peak 1 (matrine), peak 2 (oxymatrine), peak 3 (*N*-formyl cytisine), and peak 4 (*N*-acetyl cytisine). Cytisine-type alkaloids were isolated in this plant for the first time.

### 2.4. Results of HPLC-UV Analysis

**Figure 3:**
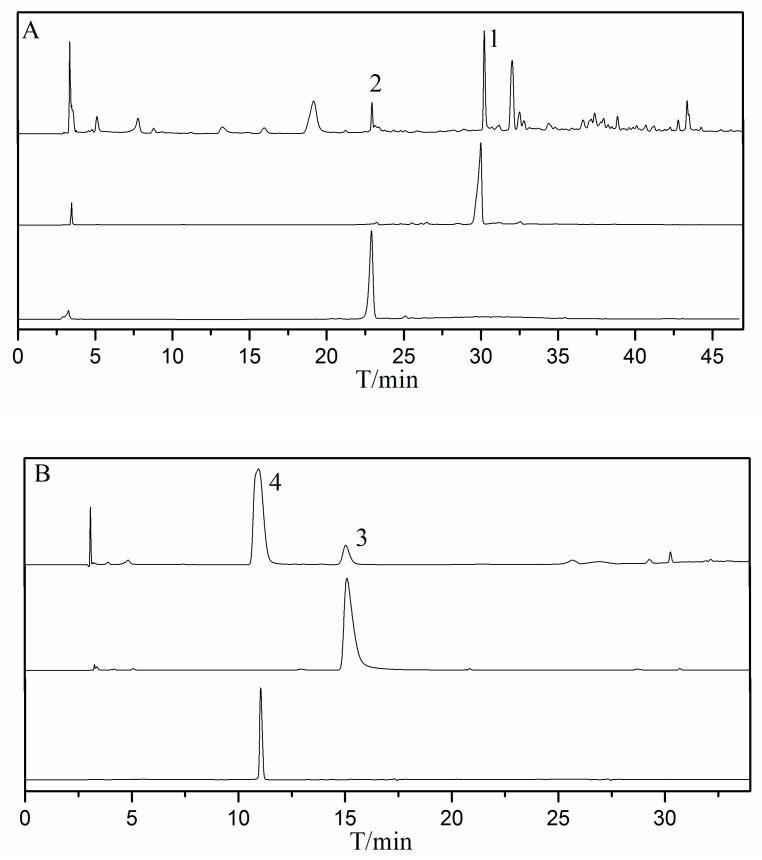
The HPLC-UV analysis of A, B, and HSCCC fractions; column: YMC-Triart C_18_ EXRS (250 mm × 4.6 mm, i.d., 5μm); mobile phase: Solvent: Distilled water (S_1_) and methanol (S_2_); gradient (A): 0.0 min (10% S_2_)–40 min (95% S_2_); gradient (B): 0.0 min (10% S_2_)–35 min (95% S_2_); flow rate: 0.8 mL/min; wavelength: 254 nm; temperature: 30°C; sampling volume: 20 μL; detector: 1260 Quat Pump VL (G1311C).

Figure 3 shows the HPLC-UV chromatograms of fraction A and B and HSCCC fractions. The purity of purified alkaloids calculated by peak area normalization method was higher than 93%.

### 2.5. Identification of Purified Alkaloids

**Figure 4:**
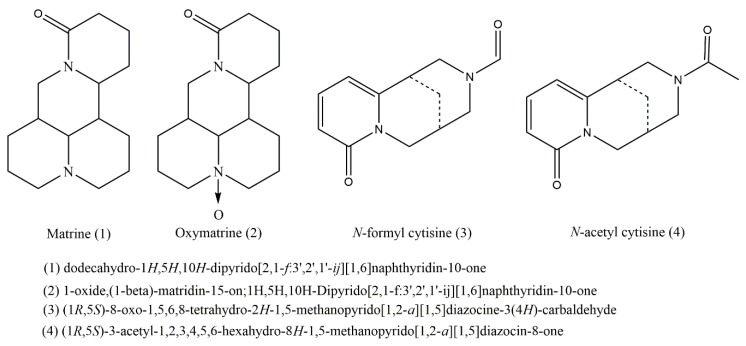
Structures of alkaloids purified by HSCCC from the stem of the *E. tubulosa* Dunn.

The structure elucidations of compounds **1**–**4** were finally achieved by analyzing the EI-MS and NMR spectroscopic data and comparing them with data from the literature [19,20,21]. Their spectral data are shown in Figure S1–S4. Each group of ^13^C-NMR of compound **3** had two sets of similar overlapping signals. Because the C-N linked to formyl groups on N is linked by a single bond, it is easy to change the configuration, and the NMR spectra show splitting [22,23]. The NMR spectra of compound **4** were almost the same as those of compound **3**. They were paired signals. However, the n-aldehyde hydrogen signal disappeared, and there was an additional methyl proton signal associated with the acyl group and a methyl carbon signal associated with the acyl group in ^13^C-NMR. Finally, compounds **1**–**4** were identified as Matrine (**1**), Oxymatrine (**2**), *N*-formyl cytisine (**3**), *N*-acetyl cytisine (**4**), respectively. Their structures are shown in Figure 4.

### 2.6. Applicability Verification Test

#### 2.6.1. Precision and Repeatability Test

The Rlative Sandard Deviation (RSD) of the four alkaloid peak areas was 1.81%, 1.59%, 2.36%, 2.17%, respectively. It shows that the precision of the instrument was good, and the experiment was reproducible.

#### 2.6.2. Applicability Test 

The higher the HSCCC temperature was, the easier the two-phase solvent system was to emulsify to get a higher S_f_. Considering that dichloromethane has a boiling point of only 39 °C, it was easy to cause solvent vaporization due to a too high temperature, which will reduce the separation effect. In combination with the use of the instrument, conditions were set to the speed of 700–900 rpm/min, a flow rate of 2–4 mL/min, and a temperature of 25 °C.

The S_f_ value was calculated according to Formula (2) and the results are shown in the Table 2. It can be found that this method has wide applicability (S_f_ > 50%).

## 3. Materials and Methods

### 3.1. Reagents and Materials

Whole plants of *E. tubulosa* Dunn. were collected from the Tianping Mountain Reserve of Badagongshan National Forest Park in Zhangjiajie, Hunan Province, China, identified by Ai-Wen Dong, research fellow, Hunan Key Laboratory of Forest Chemical Engineering, Jishou University. The voucher deposit number: YSF2017-11694. All solvents used for experiments were of analytical grade (Concord Technology Co., Ltd., Tianjin, China). Methanol used for HPLC-UV was of chromatographic grade (Concord Technology Co., Ltd., Tianjin, China). Silica gels (60–100, 100–200 mesh) used for sample preparation were purchased from the Qingdao Ocean Chemical Plant (Shanghai, China); water used was laboratory self-control distilled water. Thin-layer chromatography (TLC) was used with GF254 plates (Qingdao Marine Chemical, Inc., Shandong, China).

### 3.2. Apparatus

The instrument of HSCCC using stepwise elution was a TBE-300B high-speed counter-current chromatograph (Tauto Biotechnique, Shanghai, China) assembled by a polytetrafluoroethylene multilayer coil (i.d. of the tubing = 1.5 mm, total volume = 285 mL) and a 20 mL sample loop. The β values of the multilayer coil varied from 0.5 at the internal terminal to 0.8 at the external terminal (β = *r*/*R*, where *r* was the rotation radius from the coil to the holder shaft, and *R* was the revolution radius between the holder axis and central axis of the centrifuge or the distance). The system was also equipped with a TBP1002ST constant pump (Tauto Biotechnique, Shanghai, China), a model TBD2000-UV monitor (Tauto Biotechnique, Shanghai, China). The datum was recorded by the WH500-USB chromatography workstation (Tauto Biotechnique, Shanghai, China).

NMR experiments were carried out using a Bruker AVANCE III NMR spectrometer (600 Hz, Bruker Biospin, Germany) with CHCl_3_ (CDCl_3_) or MeOH (MeOD) as the solvent. MS analysis of purified alkaloids was performed by the EI mass spectrometer (DSQ, Thermo, USA).

### 3.3. Preparation of the Crude Sample

The dried powder (60 mesh) from the stem of *Euchresta tubulosa* Dunn. (3 kg) were extracted under reflux with 10 L of 95% industrial alcohol 3 times, at 3 hours each time, at 80 °C. Then, the extract was filtrated, combined, and vacuum evaporated at 50 °C. The residue of the mixed extract was dissolved in 0.8 L acid aqueous solution with a pH of 2.0 adjusted by 2% hydrochloric acid. The acid water solution was extracted with petroleum ether to degrease, and then the pH of the acidic aqueous solution was slowly increased to 9.5 using aqueous ammonia. Finally, the total alkaloid extract was extracted with equivalent volume trichloromethane 4 times. The trichloromethane extract was combined and vacuum evaporated at 50 °C. The crude alkaloid extract (15.2 g) was preserved at −5°C for further separation and purification.

### 3.4. Selection of the Two-Phase Solvent Systems

Total alkaloids (2.5 g) were dissolved in petroleum ether with 2.8 g 60–100 mesh silica gel and separated preliminaries with 100–200 mesh column chromatography silica-gel using a silica-gel column (26 mm × 457 mm). Gradient elution was carried out with a CH_2_Cl_2_: MeOH (1:0, 50:1, 25:1, 10:1, 0:1, *v*/*v*) solvent system (all adding triethylamine to adjust pH 7–8). Each 80 mL eluent was a fraction. TLC was used to track and detect each fraction. According to the R_f_ of the eluent, 2 fractions A (0.7 g) and B (1.1 g) were found by merging their similar products. Other fractions were of less quality and more complex compounds, thus this study did not consider them.

Various types of two-phase solvent systems composed of CCl_4_-CH_2_Cl_2_-MeOH-H_2_O with different volume ratios were equipped with a small volume (8 mL) and completely stratified by ultrasound. An equivalent volume (2 mL) of the upper and lower phases of the 7 2-phase solvent systems was absorbed to make a small solvent system, and then 1 mg of crude alkaloid extracts was dissolved in the small solvent system. The *K* of the single alkaloid was calculated by HPLC-UV and the formula (1):

*K* = *A*_s_/*A*_m_ (1)

*A*_s_: Peak area of a single alkaloid in the stationary phase;
*A*_m_: Peak area of a single alkaloid in the mobile phase.

### 3.5. Preparation of HSCCC Two-Phase Solvent Systems and Sample Solution

The final 2-phase solvent systems CCl_4_-CH_2_Cl_2_-MeOH-H_2_O (2:3:3:2, *v*/*v*) and CH_2_Cl_2_-MeOH-H_2_O (5:3:2, *v*/*v*) were prepared in a separatory funnel separately and stratified at normal temperature completely. Then, the blister of the upper phase and the lower phase was exhausted by ultrasonic for 25 min before use. The sample (300 mg) was dissolved in a mixed solution of 5 mL of each phase of CCl_4_-CH_2_Cl_2_-MeOH-H_2_O (2:3:3:2, *v*/*v*) to prepare the sample solution.

### 3.6. HSCCC Separation Procedures

Before the beginning of the experiment, the multilayer helical column was completely filled with the upper phase of carbon tetrachloride-methylene chloride-methanol-water (2:3:3:2, *v*/*v*) at a flow rate of 15 mL/min. Then, the ultraviolet detector was turned on at 254 nm. The instrument was run at a revolution speed of 900 rpm and the constant temperature tank was opened at 25 °C. At the same time, the lower phase of the carbon tetrachloride-methylene chloride-methanol-water (2:3:3:2, *v*/*v*) was pumped into the chromatographic column at a speed of 1.8 ml/min of the head-tail elution mode until the liquid effluent formed a stable 2-phase system. The sample (300 mg) was injected into the column through the injection valve when hydrodynamic equilibrium was established. Fractions were manually collected per 10 mL according to the spectrum of the ultraviolet detector. The pump was stopped at about 170 min and the mobile phase was changed to the lower phase of methylene chloride-methanol-water (5:3:2, *v*/*v*) at 1.8 mL/min. The compounds were collected according to the UV detector. Then, samples were stored at −5 °C and merged by subsequent HPLC-UV analysis. Finally, all the instruments were closed at about 300 min and the liquid in the column was blown clean with nitrogen gas to recover the sample. The S_f_ was 72%.

### 3.7. HPLC-UV Analysis 

The sample was analyzed by HPLC-UV (Agilent 1260, Agilent Technologies Inc., USA) with a YMC-Triart C_18_ EXRS (250 mm × 4.6 mm, i.d., 1.5 mm); mobile phase: Distilled water (S_1_) and methanol (S_2_); temperature: 30 °C; flow rate: 0.8 mL/min; injection volume: 20 μL; wavelength: 254 nm; elution conditions (A): 0.0 min (10% S_2_)–45 min (95% S_2_); elution conditions (B): 0 min (10% S_2_)–35 min (95% S_2_); detector: 1260 Quat Pump VL (G1311C, Agilent Technologies Inc., USA).

### 3.8. Applicability Verification Test

#### 3.8.1. Precision and Repeatability Test

The HSCCC sample obtained by liquid-liquid extraction of total alkaloids was repeatedly injected for 6 times according to the method in 3.6., with 300 mg each time. The peak areas of target compounds were recorded separately.

#### 3.8.2. Applicability Test

The S_f_ was a very important parameter in HSCCC, and the feasibility of the operating parameters depended on S_f_. In this experiment, the effects of speed, temperature, and flow rate on S_f_ were investigated. A higher S_f_ value can obtain a better separation effect of HSCCC.

In this verification experiment, the upper phase of the 2-phase system (1) was filled with the whole pipeline at 15mL/min (the outlet of the pipeline was connected to a 250 mL measuring cylinder). Then, the lower phase of the system (1) was pumped into the instrument. The volume (*V*_1_) of the upper phase in the measuring cylinder was recorded until the liquid outflow at the outlet of the pipe formed a 2-phase and the baseline of the UV detector was balanced. Then the mobile phase was replaced with the lower phase of the system (2). The volume (*V*_2_) of the upper phase in the measuring cylinder was recorded until the liquid outflow at the outlet of the pipe formed a 2-phase and the baseline of the UV detector was balanced. Finally, all the liquid in the instrument was blown out to the measuring cylinder, and the volume (*V*_3_) of the upper phase retained in the instrument was recorded.

S_f_ = *V*_3_/(*V*_1_ + *V*_2_ + *V*_3_) (2)

*V*_1_: Loss volume of the first phase separation;
*V*_2_: Loss volume of the second phase separation;
*V*_3_: Volume of upper phase retained in the instrument.

## 4. Conclusions

Four alkaloids matrine (**1**), oxymatrine (**2**), *N*-formyl cytisine (**3**), and *N*-acetyl cytisine (**4**) were isolated and purified systematically by HSCCC using stepwise elution. Cytisine-type alkaloids were isolated for the first time from this plant by this mode. The results of the research provided a successful pattern for isolation and purification of alkaloids with different polarities. In this mode, only the liquid-liquid extraction and once the HSCCC using stepwise elution were used to obtain four alkaloids. It can make compounds separated quickly and effectively. This mode also solves the problem that acid or ionic solution needs to be added to obtain suitable *K*, which is difficult to deal with in the later stage, adsorption on the solid carriers, and separation of alkaloids with different polarities. The verification test (S_f_ > 50%) of this mode shows that it has wide applicability.

## Figures and Tables

**Table 1:** *K* values of the target compounds in two-phase solvent systems.

Solvent Systems	Volume Ratio (*v*/*v*)	*K*_1_	*K*_2_	*K*_3_	*K*_4_
CH_2_Cl_2_-MeOH-H_2_O	2: 1: 1	0.23	0.53	0.51	0.68
	5: 3: 2	0.25	0.56	0.65	0.82
	4: 3: 2	0.31	0.63	0.77	0.98
CCl_4_-CH_2_Cl_2_-MeOH-H_2_O	1: 4: 3: 2	0.38	0.81	-	-
	2: 3: 3: 2	0.76	0.95	-	-
	3: 2: 3: 2	0.92	1.89	-	-
	4: 1: 3: 2	2.33	4.43	-	-

**Table 2:** The effect of the flow rate and revolution speed on the retention of the stationary phase.

Rotation Speed (rpm)	Flow Velocity (mL/min)	S_f_	Rotation Speed (rpm)	Flow Velocity (mL/min)	S_f_
700	2	0.645	800	3	0.657
700	2.5	0.638	800	3.5	0.626
700	3	0.614	800	4	0.587
700	3.5	0.581	850	2	0.702
700	4	0.553	850	2.5	0.683
750	2	0.662	850	3	0.671
750	2.5	0.643	850	3.5	0.641
750	3	0.639	850	4	0.615
750	3.5	0.609	900	2	0.723
750	4	0.568	900	2.5	0.698
800	2	0.675	900	3	0.682
800	2.5	0.669	900	3.5	0.654
			900	4	0.639

## Supplementary Materials

The following are available online, Figure S1: Analysis of compound **1**, Figure S2: Analysis of compound **2**, Figure S3: Analysis of compound **3**, Figure S4: Analysis of compound **4**.
