# Supplementary material for: Preparative Separation of Alkaloids from Stem of Euchresta tubulosa Dunn. by High-Speed Counter-Current Chromatography Using Stepwise Elution
Source: Molecules. 2019 Dec 16;24(24):4602. doi: 10.3390/molecules24244602 (PMC6943640; doi:10.3390/molecules24244602)
Supplement: Supplementary file 1 [file molecules-24-04602-s001.pdf]

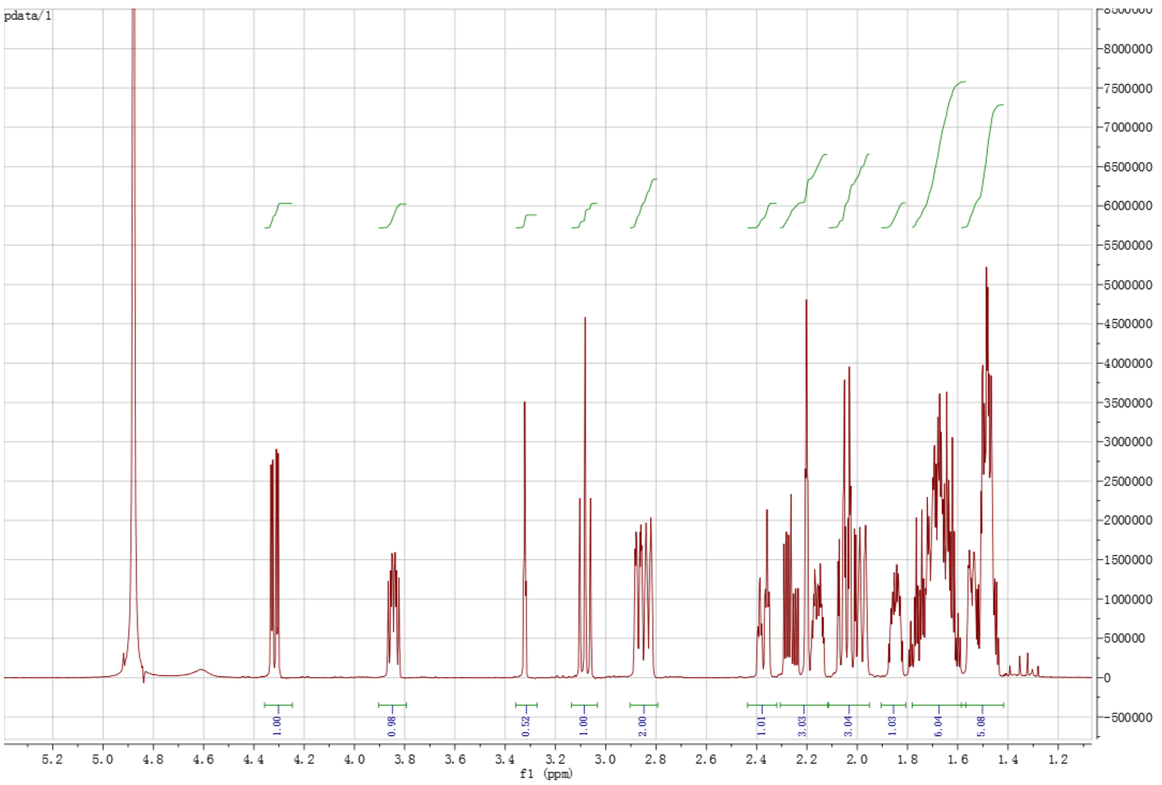
pdata/1
1.00
0.98
0.52
1.00
2.00
1.01
3.03
3.04
1.03
6.04
5.08
f1 (ppm)

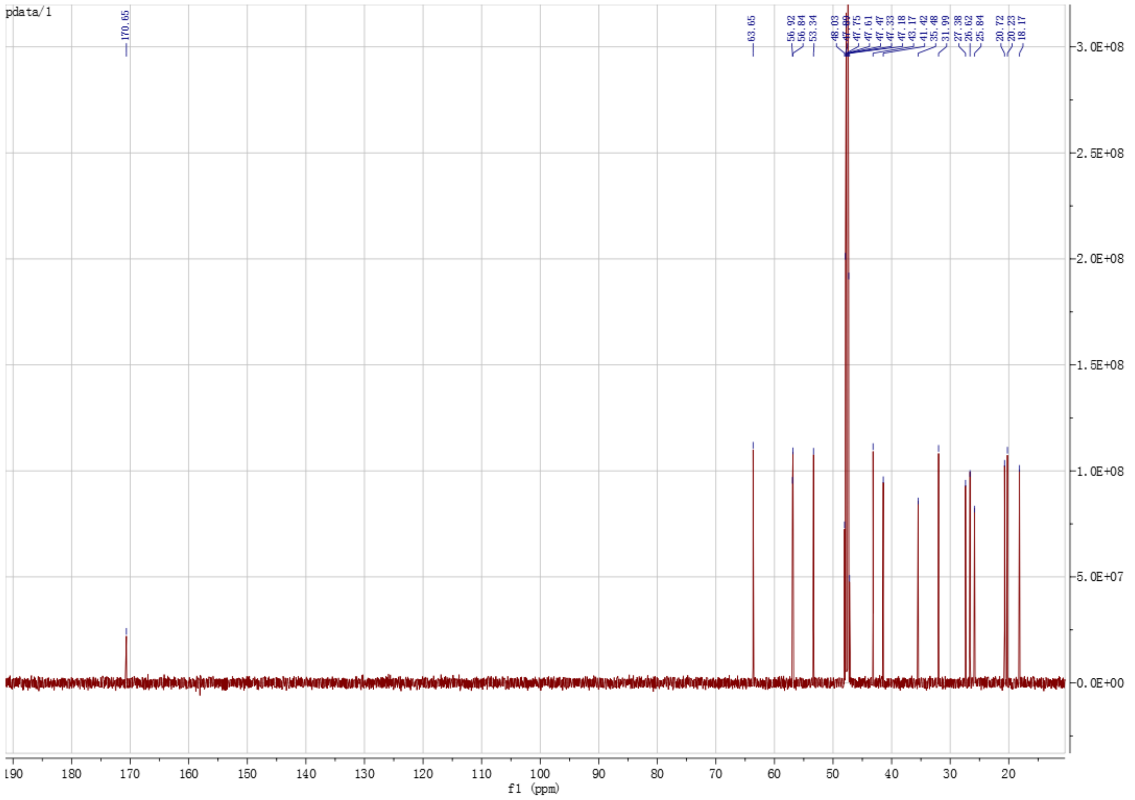
pdata/1
170.65
63.65
56.92
56.84
53.34
f1 (ppm)

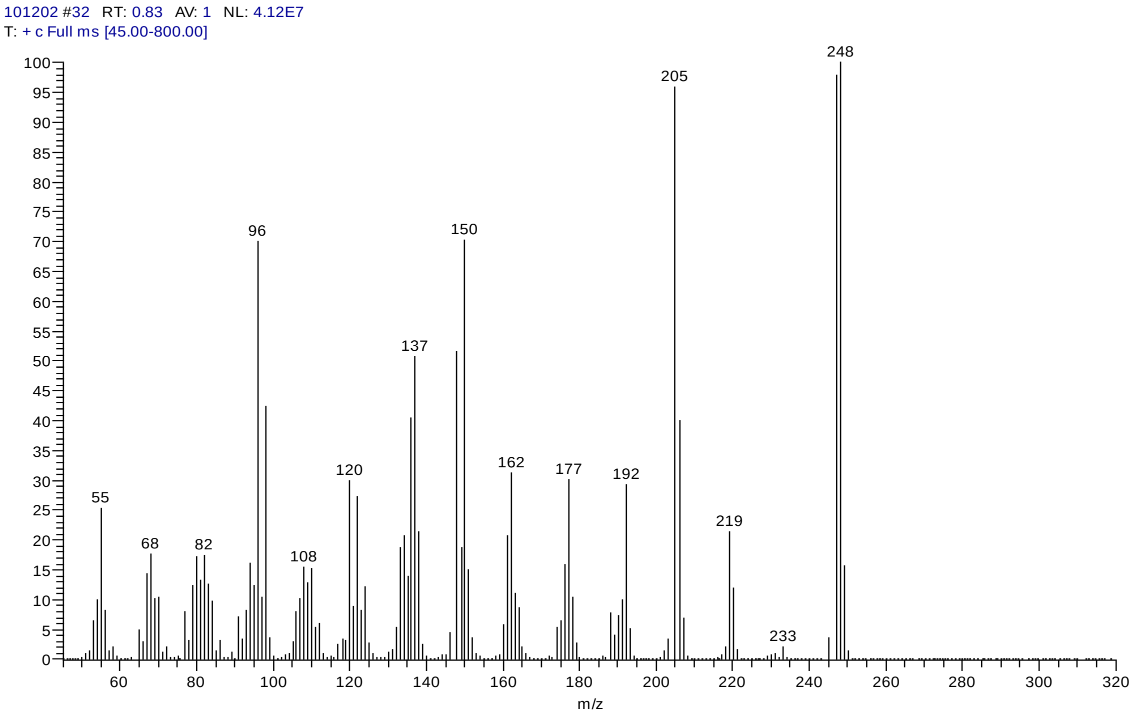

Figure S1. Analysis of compound 1.

Matrine (peak 1 in Fig. 2): EI-MS, *m/z* 248 [M]+; ^{1}H-NMR (600MHz, MeOD) δ: 2.91-2.85 (m, 1H, H-2), 2.05-2.00 (m, 1H, H-2'), 1.80–1.56 (m, 6H, 3, 4, 5, 8, H-12, H-13'), 1.56-1.42 (m, 5H, H-3', 4', 8', 9', H-7), 2.20 (m, 1H, H-6), 2.20-2.12 (m, 1H, H-9), 2.85–2.79 (m, 1H, H-10), 2.11-2.05 (m, 1H, H-10'), 3.84 (ddd, 1H, *J*=10.9, 8.9, 5.8 Hz, H-11), 1.89-1.80 (m, 1H, H-12'), 2.00–1.94 (m, 1H, H-13), 2.37 (dtd, 1H, *J*=17.6, 4.7, 1.5 Hz, H-14), 2.26 (ddd, 1H, *J*=17.0, 10.7, 5.5 Hz, H-14'), 4.32 (dd, 1H, *J*=12.8, 4.3 Hz, H-17), 3.08 (t, 1H, *J*=12.8Hz, H-17'). ^{13}C-NMR (151MHz, MeOD) δ: 56.92(N-CH_2, C-2), 20.23 (CH_2, C-3), 26.62 (CH_2, C-4), 35.48 (CH, C-5), 63.65 (N-CH, C-6), 41.42 (CH, C-7), 25.84 (CH_2, C-8), 20.72 (CH_2, C-9), 56.84 (N-CH_2, C-10), 53.34 (N-CH, C-11), 27.38 (CH_2, C-12), 18.17 (CH_2, C-13), 31.99 (CH_2, C-14), 170.65 (N-C=O, C-15), 43.17 (N-CH_2, C-17).

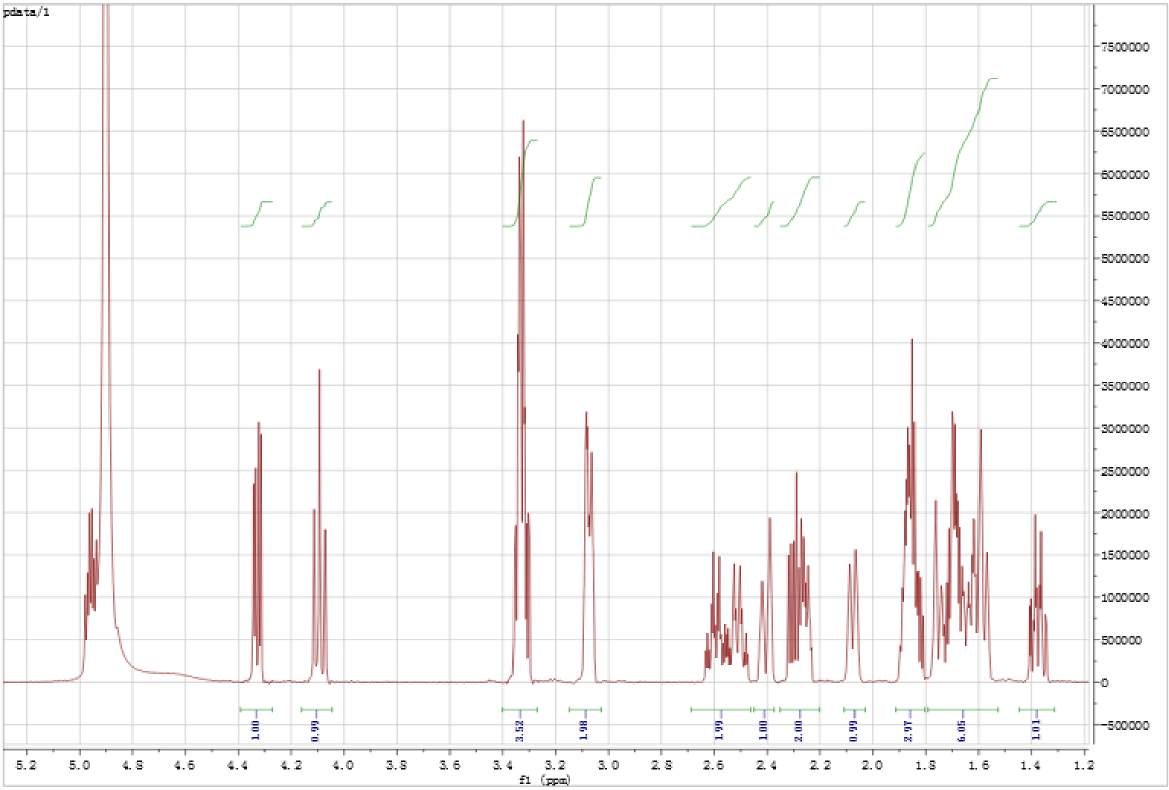
pdata/1
f1 (ppm)

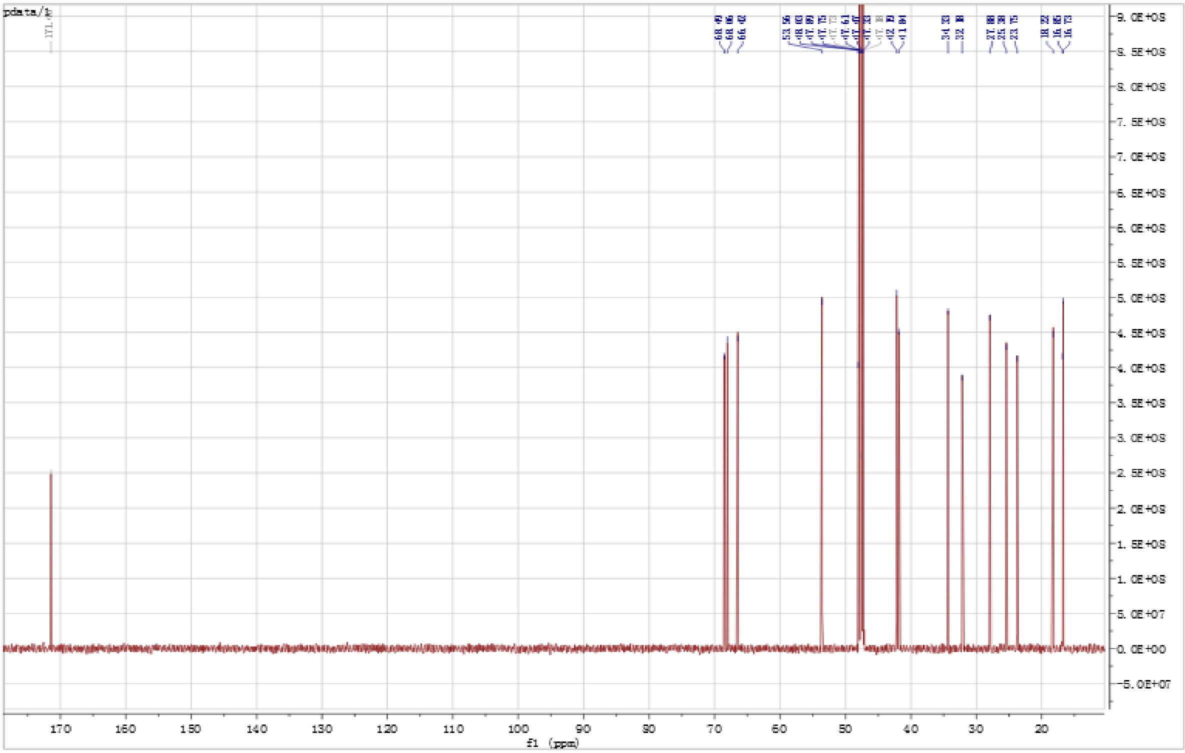
pdata/1
f1 (ppm)

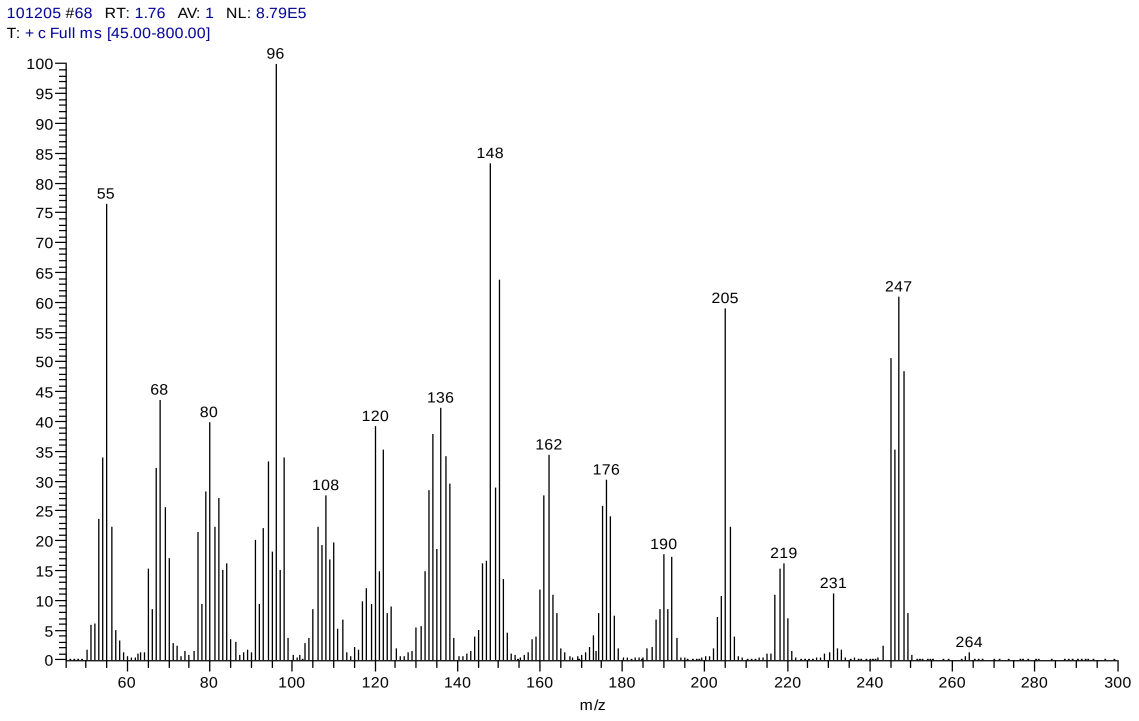

Figure S2. Analysis of compound 2.

Oxymatrine (peak 2 in Fig. 2): EI-MS, *m/z* 264 [M]+; ^{1}H-NMR (600MHz, MeOD) δ: 3.39-3.28 (m, 3H, 2, 10, H-11), 3.07 (ddd, 2H, *J*=10.2, 4.4, 2.2 Hz, H-2', 10'), 2.65-2.44 (m, 2H, CH_2-3), 1.91-1.80 (m, 3H, H-4, 5, 8), 1.80-1.54 (m, 4H, H-4', 7, 8, 9'), 4.96 (td, 1H, *J*=10.0, 5.5 Hz, H-6), 2.08 (dt, 1H, *J*=12.9, 4.2Hz, H-9), 2.34-2.22 (m, 2H, H-12', 14'), 1.37 (tdd, 1H, *J*=12.7, 9.5, 3.1Hz, H-12), 1.54 (m, 2H, CH_2-13), 2.44-2.36 (m, 1H, H-14), 4.33 (dd, 1H, *J*=12.2, 5.0 Hz, H-17), 4.09 (t, 1H, *J*=12.5Hz, H-17'). ^{13}C-NMR (151MHz, MeOD) δ: 68.06 (N-CH_2, C-2), 16.85 (CH_2, C-3), 25.37 (CH_2, C-4), 34.33 (CH, C-5), 68.50 (N-CH, C-6), 41.84 (CH, C-7), 23.75 (CH_2, C-8), 18.21 (CH_2, C-9), 66.42 (N-CH_2, C-10), 53.56 (N-CH, C-11), 27.88 (CH_2, C-12), 16.73 (CH_2, C-13), 32.18 (CH_2, C-14), 171.40 (N-C=O, C-15), 42.19 (N-CH_2, C-17).

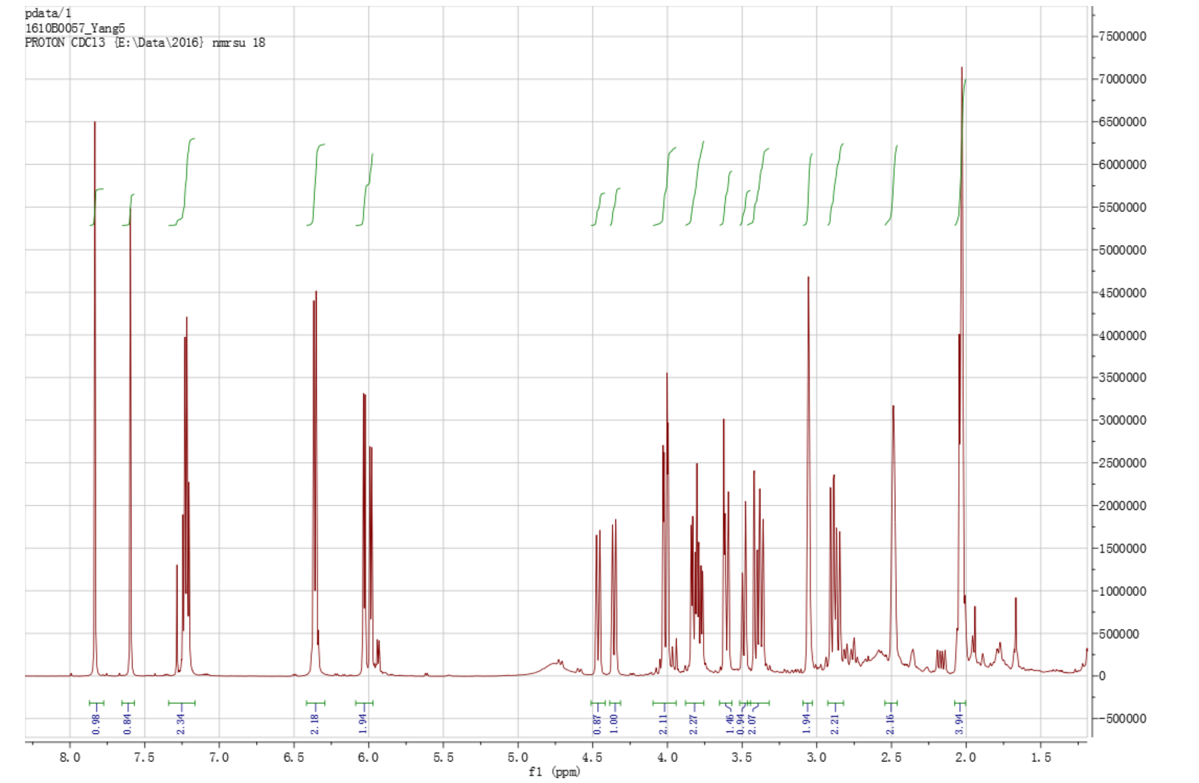
pdata/1
1610B0057_Yang5
PROTON CDCl3 {E:\Data\2016} nmrsu 18
0.98
0.84
2.34
2.18
1.94
0.87
1.00
2.11
2.27
1.46
0.94
2.07
1.94
2.21
2.16
3.94
f1 (ppm)

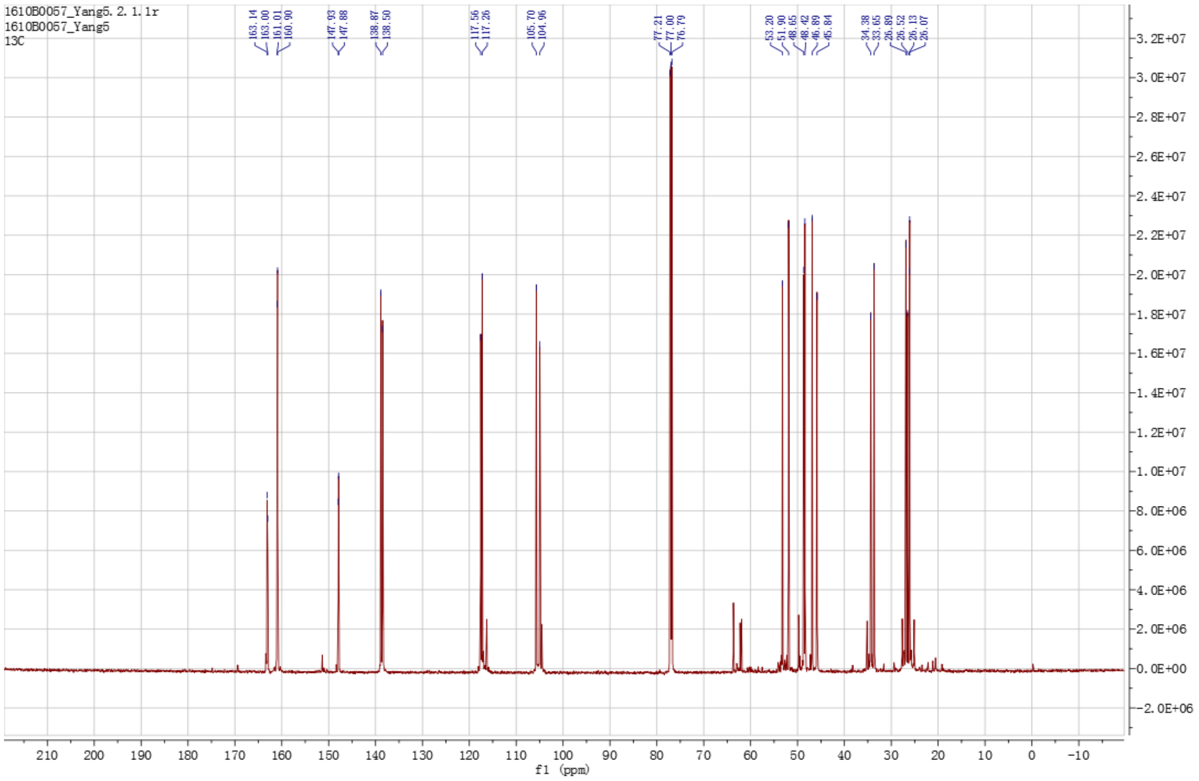
1610B0057_Yang5.2.1.1r
1610B0057_Yang5
13C
163.14
163.00
161.01
160.90
147.93
147.88
138.87
138.50
117.56
117.26
105.70
104.96
77.21
77.00
76.79
53.20
51.90
48.65
48.42
46.89
45.84
34.38
33.65
26.89
26.52
26.13
26.07
f1 (ppm)

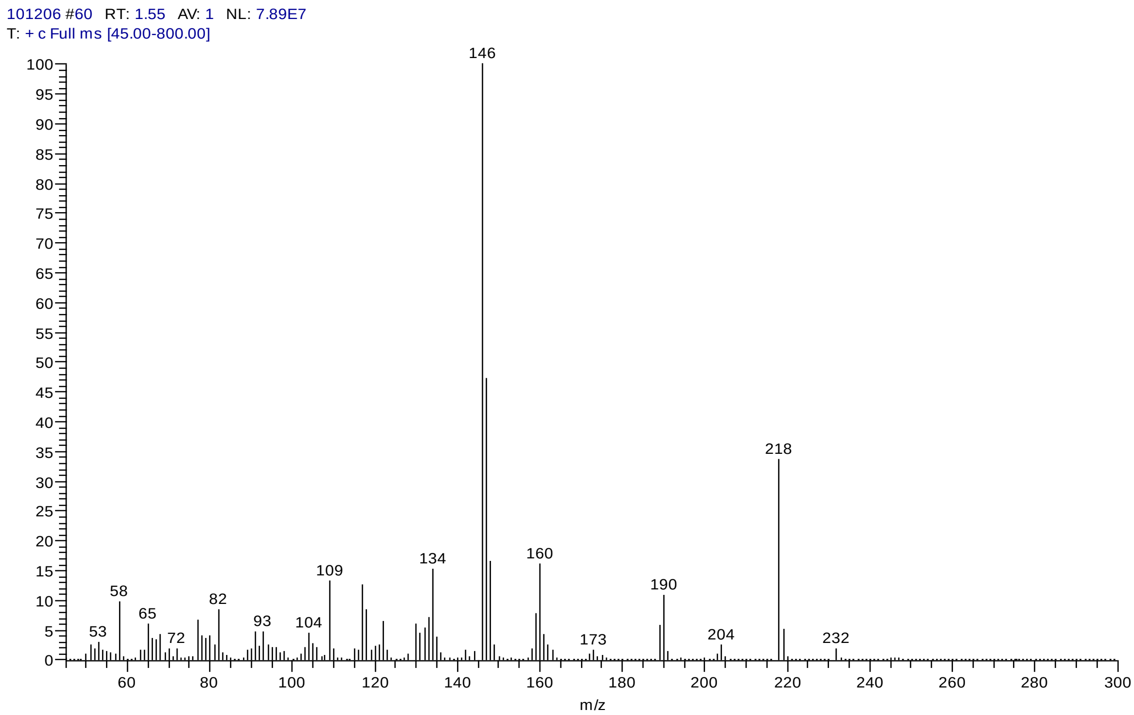

Figure S3. Analysis of compound 3.

N-formyl cytisine (peak 3 in Fig. 2): EI-MS, *m/z* 218 [M]+; ^{1}H-NMR (600MHz, $CDCl_3$) δ: 3.44/ 3.42 (d/dd, 1H, *J*= 12.8, 2.5 Hz, H-2), 3.56/ 3.53 (d, 1H, *J*=1.8 Hz, H-2'), 2.55 (1H, H-3), 3.85/ 3.88 (dd, 1H, *J*=15.7, 6.0 Hz, H-4), 4.10/ 4.08 (d, 1H, *J*=15.6, 3.6 Hz, H-4'), 6.45/ 6.44 (d, 1H, *J*=9.0 Hz,H-7), 7.27/ 7.19 (m, 1H, H-8), 6.08/ 6.02 (d, 1H, *J*=6.8Hz, H-9), 3.10 (m, 1H, H-11), 4.46/ 4.36 (d, 1H, *J*=13.6Hz, H-12), 2.96/ 2.92 (dd/d, 1H, *J*=13.2, 1.2Hz, 15.0Hz, H-12'), 2.08/ 2.09 (dd, 2H, *J*=8.3, 4.8Hz, CH_2-13), 7.83/7.60 (s, 1H, H-14). ^{13}C-NMR (151MHz, $CDCl_3$) δ: 51.90/ 45.84 (N-CH_2, C-2), 26.89/ 26.52 (CH, C-3), 48.65/ 48.42 (N-CH_2, C-4), 163.14/ 163.00 (N-C=O, C-6), 117.56/ 117.26 (CH, C-7), 138.87/ 138.50 (CH, C-8), 104.96/ 105.70 (CH, C-9), 147.93/ 147.88 (C, C-10), 33.65/ 34.38 (CH, C-11), 53.20/ 46.89 (N-CH_2, C-12), 26.13/ 26.07 (CH_2, C-13), 160.90/ 161.01 (N-CHO, C-14).

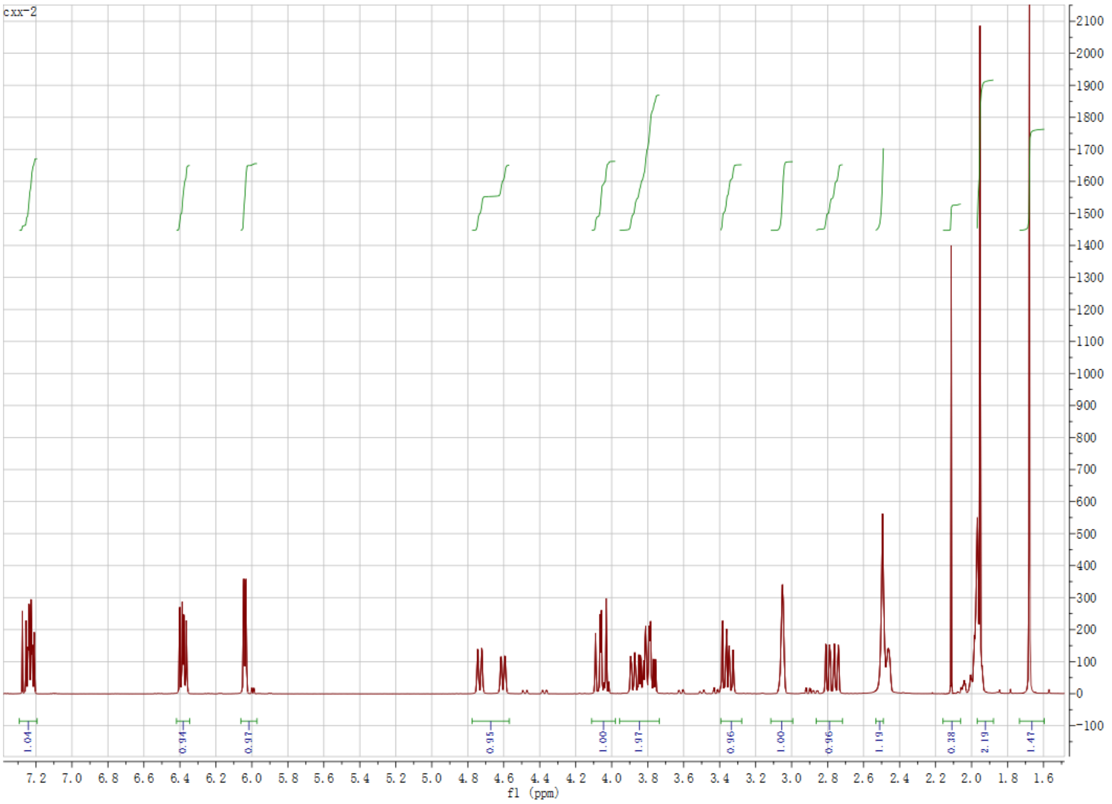
cxx-2
1.04
0.94
0.97
0.95
1.00
1.97
0.96
1.00
0.96
1.19
0.38
2.19
1.47
f1 (ppm)

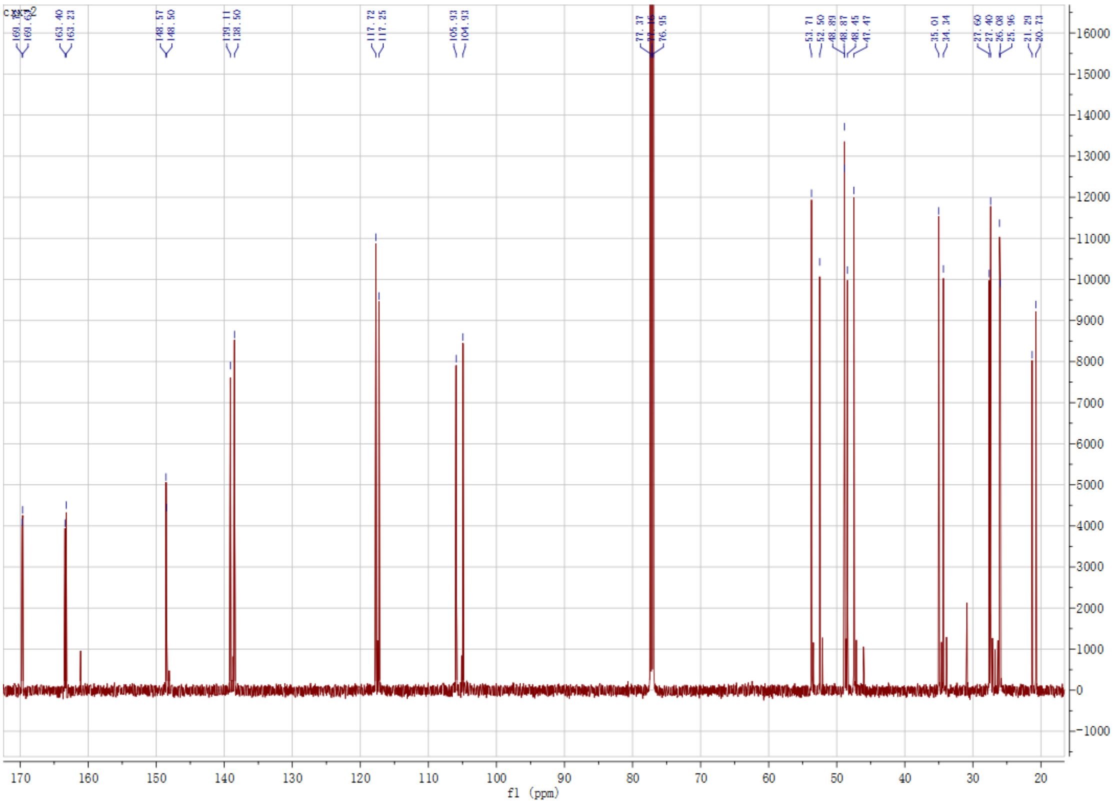
163.40
163.23
148.57
148.50
139.11
138.50
117.72
117.25
105.93
104.93
77.37
76.95
53.71
52.50
48.45
47.47
35.01
34.34
27.60
27.40
26.08
25.96
21.29
20.73
f1 (ppm)

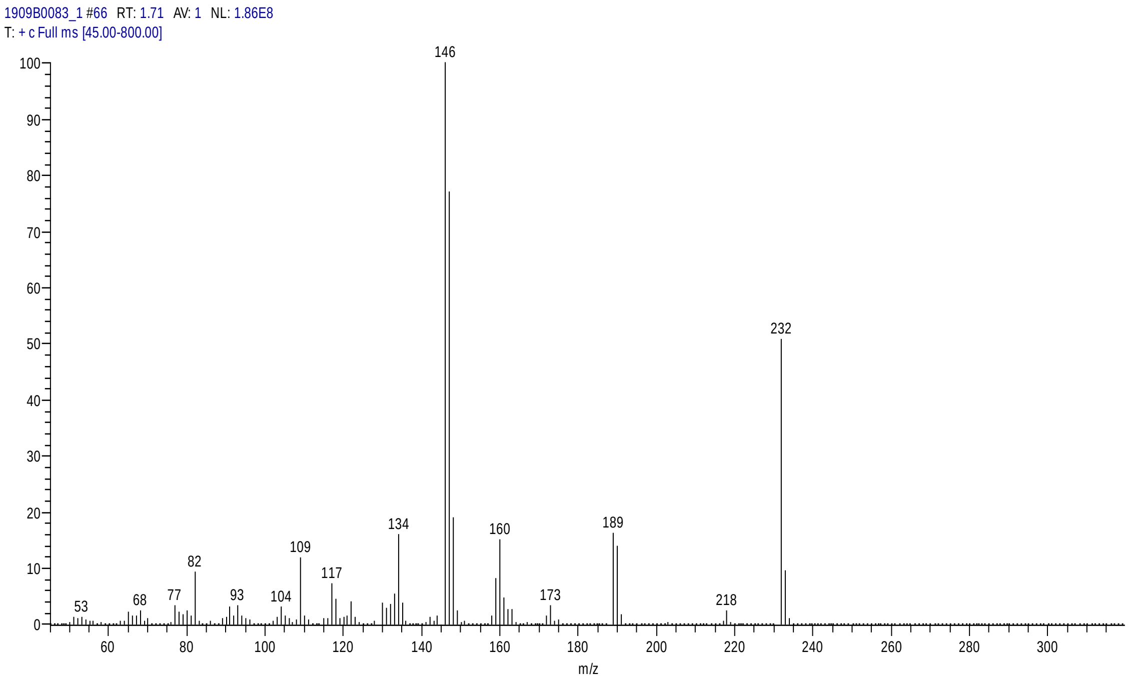

Figure S4. Analysis of compound 4.

N-acetyl cytisine (peak 4 in Fig. 2): EI-MS, *m/z* 232 [M]+; ^{1}H-NMR (600MHz, $CDCl_3$) δ: 1.68 (s, 2H, CH_2-13), 1.98 (s, 3H, CH_3-15), 2.48 (s, 1H, H-3), 2.84 (m, 1H, H-11), 3.05 (s, 1H, H-4), 3.34 (m, 1H, H-4), 3.72-3.89 (m, 2H, CH_2-2), 4.01/ 4.07 (d, 1H, *J*=10.1Hz, H-12), 4.60/ 4.72 (d, 1H, *J*=13.1Hz, H-12), 6.03/ 6.04 (d, 1H, *J*=6.8Hz, H-9), 6.36 (m, 1H, H-7), 7.25 (m, 1H, H-8). ^{13}C-NMR (151MHz, $CDCl_3$): δ: 21.29/ 20.73 (CH_3, C-15), 26.08/ 25.96 (CH, C-3), 27.60/ 27.40 (CH_2, C-13), 35.01/ 34.34 (CH, C-11), 48.45/ 47.47 (N-CH_2, C-12), 48.88 (N-CH_2, C-2), 53.71/ 52.50 (N-CH_2, C-4), 105.93/ 104.93 (CH, C-9), 117.77/ 117.25 (CH, C-7), 139.11/ 138.50 (CH, C-8), 148.53 (C, C-10), 163.40/ 163.23 (N-C=O, C-6), 169.71/ 169.63 (N-C=O, C-14).
